# Uveitis update: An overview by the society for Ophthalmo-Immunoinfectiology in Europe

**DOI:** 10.4103/0974-9233.58413

**Published:** 2009

**Authors:** Carl P Herbort

**Affiliations:** Retinal and Inflammatory Eye Diseases, Centre for Ophthalmic Specialised Care & University of Lausanne, Lausanne, Switzerland

This issue of the MEAJO journal mainly containing original and educational articles on uveitis is compiled from the uveitis course and symposium organised during the last MEACO meeting in Bahrain in March 2009 by the Society for Ophthalmo-Immunoinfectiology in Europe (SOIE). Although the course was held on the last day of the meeting and consisted of a full day course, sustained attendance indicated that considerable interest exists in the Middle-East for uveitis. Together with Ahmed Abu El Asrar, the local organiser of the SOIE course and Deepak P. Edward, the Chief-Editor of MEAJO, we decided to publish a theme based issue on uveitis.

SOIE is an association oriented towards educational activities in the field of uveitis in Europe but is also organising activities beyond Europe, especially in the Middle East and North Africa. The society has organized educational courses in Saudi Arabia in 2005 and 2008 and in Bahrain in 2009. SOIE symposia were held in Krakau, Poland in 2007, in Ancona, Italy in 2008, in Cappadoccia, Turkey in 2008 as well as in Tunisia in November 2009, with significant attendance at each venue. Our group is willing to collaborate with any local partner to organise courses or symposia on uveitis. We are interested in working interactively and exchange information with the local organisers and ophthalmologists and provide educational content on the practical aspects of uveitis taking into account local specificities. We are open to all viable propositions to promote the recognition and management of uveitis and ophthalmic infectious disease.

For this MEAJO issue, the instructors of the SOIE Bahrain course were asked to contribute articles either on original ongoing uveitis research or to summarize their course presentations for didactic and educational purposes in a manuscript. A significant number of instructors made contributions so that an attractive theme oriented issue on uveitis could be produced.

In his tutorial article on the differential diagnosis of anterior uveitis Carl P. Herbort gives a practical method for the appraisal and work-up of anterior uveitis based on clinical examination. He emphasizes the simplicity of the diagnostic steps of uveitis cases as long as investigations are based on clinical signs and not on random endless lists that are published in uveitis textbooks.

The article on angiography in uveitis puts forward a comprehensive approach of angiographic investigation indicating the need for dual fluorescein and indocyanine green angiography (ICGA) in most cases where performing angiography is deemed necessary. By demonstrating lesions not shown by any other means, ICGA shows its pre-eminence over fluorescein angiography that merely gives more precision on superficial fundus lesions and RPE changes usually already identified by fundus observation, optical coherence tomography or other means.

The two articles by Professor Ahmed Abu El Asrar and his group from Riyadh touch upon two important topics in uveitis. The first article has the merit to reassess the role of tuberculosis in present day uveitis. The second article is gives an excellent summary on the difficult clinical question of retinal vasculitis. The article includes a practical approach to the diagnosis of retinal vasculitis and provides precious information for the clinician.

Who better than Professor Ilknur Tugal-Tutkun from Istanbul University could write a review article on Behçet's uveitis? This reknowned specialist who is recognized globally for her expertise in ocular Behçet's disease has put all her experience and expertise to give an overview on what the clinician should know about Behçet's uveitis. In this article the author also presents the latest developments in the management of Behçet's uveitis including laser flare photometry which has become an essential tool for non invasive follow-up of intraocular inflammation in most uveitis entities.

We have the opportunity to publish a very important and interesting review article on emergent causes of infectious uveitis; thanks to Professor Khairallah from Monastir University in Tunisia who has been at the origin of several reports on emerging agents causing uveitis. He certainly is the most appropriate person to write this report on rare and unknown etiologies of uveitis which should nevertheless be known by all practicing ophthalmologists in this era of increased travel and migration.

The last review article relates to inflammatory choroidal neovascularization, a complication of uveitis which is of rare occurence but that however has devastating consequences on visual function. Piergiorgio Neri, being both a specialist in medical retina as well as in uveitis, the review he and his team are presenting here is based on a very longstanding experience in the management of this feared complication of uveitis. So far treatment has been unsatisfactory but the availability of intra-vitreal anti-VEGF therapies seems to have drastically changed the outcome of inflammatory CNV although this statement still relies on an accumulation of cases as the authors indicate. In such rare diseases however, where controlled studies are impossible, medical treatment should be based on common sense which in this case points to the use of anti-VEGF treatment.

The theme based part of this issue is completed by an original article on Fuch's uveitis, the publication of which is especially appropriate in the MEAJO journal, as the readership mainly comes from areas where irises are predominantly brown. This article indicates that heterochromia should not be considered as a preponderant feature. Indeed in brown iris geographical areas heterochromia will rarely be seen and iris texture changes should be evaluated instead. The authors therefore recommend abandoning the term Fuch's heterochromic cyclitis and to resort to the simple eponym of Fuchs' uveitis. The main message of this article however is the importance and relevance of posterior segment involvement that has been scotomized in the last decades although it was clearly described in Ernst Fuchs' initial reports. The failure to associate posterior signs such as vitritis with Fuchs' uveitis was identified as the single most important reason for misdiagnosis or delay in the diagnosis of Fuchs' uveitis.

It is always a great pleasure for the SOIE to collaborate with our colleagues from the Middle-East where several successful projects have been conducted so far. We hope that the present project resulting in this theme based issue will be appreciated and are looking forward to future collaborations and projects.

